# Environmental enrichment prevents the late effect of acute stress-induced fear extinction deficit: the role of hippocampal AMPA-GluA1 phosphorylation

**DOI:** 10.1038/s41398-020-01140-6

**Published:** 2021-01-05

**Authors:** Leonardo Santana Novaes, Letícia Morais Bueno-de-Camargo, Carolina Demarchi Munhoz

**Affiliations:** Department of Pharmacology, Universidade de Sao Paulo Instituto de Ciencias Biomedicas, São Paulo, 05508-000 Brazil

**Keywords:** Molecular neuroscience, Hippocampus

## Abstract

The persistence of anxiety and the deficit of fear memory extinction are both phenomena related to the symptoms of a trauma-related disorder, such as post-traumatic stress disorder (PTSD). Recently we have shown that single acute restraint stress (2 h) in rats induces a late anxiety-related behavior (observed ten days after stress), whereas, in the present work, we found that the same stress impaired fear extinction in animals conditioned ten days after stress. Fourteen days of environmental enrichment (EE) prevented the deleterious effect of stress on fear memory extinction. Additionally, we observed that EE prevented the stress-induced increase in AMPA receptor GluA1 subunit phosphorylation in the hippocampus, but not in the basolateral amygdala complex and the frontal cortex, indicating a potential mechanism by which it exerts its protective effect against the stress-induced behavioral outcome.

## Introduction

Long-lasting symptoms of anxiety are a recurring condition in people experiencing stressful situations and are causally related to the symptomatology of post-traumatic stress disorder (PTSD). A single 2-h restraint stress session is sufficient to generate an anxious-related behavior in rats that persists for at least ten days^1,2^. In addition to the persistence of anxiety, impairment of the fear memory extinction is also a characteristic symptom in people with PTSD^3^.

The activation of inhibitory brain circuits that reduce the expression of fear governs fear memory extinction^4^, which is one of the neurobiological bases of cognitive-behavioral therapy. This intervention had proven to be effective in patients with PTSD^5^. The extinction of conditioned fear is considered an active process of learning a new inhibitory association^6,7^, and, therefore, requires molecular changes related to neural plasticity^8^. The circuitry composed of the prefrontal cortex (PFC), the basolateral complex of the amygdala (BLA), and the hippocampus (HC) is vital for mediating this phenomenon^7^. Importantly, these brain structures undergo adaptive, plastic changes after acute or chronic exposition to stress^9^.

Contextual evidence profoundly influences extinction memory. It is widely accepted that the HC sends conceptual representations of the context to the BLA and PFC, whose reciprocal connections are critical to the behavioral outcome triggered by the footshock paired stimulus^10^. Substantial evidence suggests that the modification in the phosphorylation state and surface expression of some AMPA glutamate receptor subunits (as glutamate subunit 1, GluA1) in the HC critically influences the proper fear extinction^11^.

Several approaches have been used to identify predisposing and protective factors related to the behavioral response to stressors. Environmental enrichment (EE) is an experimental, non-pharmacological model widely used for this purpose, with well-known anxiolytic-like properties^12^. Recently, we found that EE can prevent the immediate and late (observed 10 days after stress) effects of acute restraint stress on anxiety-like behavior in rats^2,13^. However, it is still poorly known the effect of both EE and this type of stress on the extinction of aversive memory. To further explore this, in the present work, we sought to explore the late effects of acute restraint stress on fear memory extinction, the role of the previous exposition to EE in this scenario, and the potential plasticity-related molecular changes in the HC, BLA, and frontal cortex (FC).

## Materials and methods

### Subjects

A total of 56 male Wistar rats (60 days old at the beginning of the experiments) from the Facility for SPF Rat Production at the Institute of Biomedical Sciences—Animal Facility Network at the University of São Paulo and maintained on the Facility of Pharmacology Department—Unit I was used as experimental subjects. All the experiments were conducted under the standards of the Ethics Committee for Animal Use of the Institute of Biomedical Sciences/University of São Paulo (CEUA-ICB 85/2016) and the guidelines of the Brazilian National Council for the Control of Animal Experimentation (CONCEA).

Animals were randomly pair-housed in groups in standard polypropylene cages (30 × 40 × 18 cm) for a period of 7–10 days (habituation period), after which they were distributed according to the experimental group. Animals were housed with free access to water and food, kept in a light–dark cycle of 12 h (lights on at 07:00 a.m.), and under controlled temperature (23 ± 2 °C).

We took all efforts to minimize animal suffering and reduce the number of animals to the minimum required to detect significant statistical effects.

### Experimental design

**Fig. 1:**
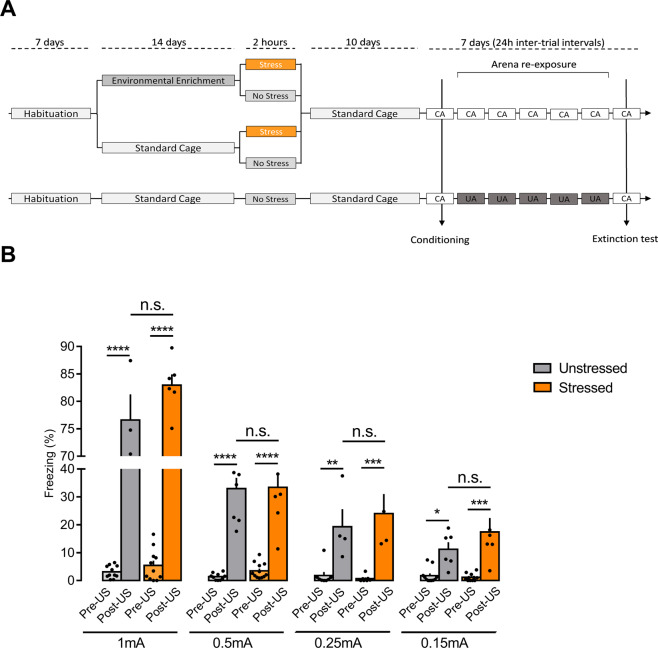
Experimental design and the stress influence on the acquisition of contextual fear memory. Schematic timeline of the experimental procedure (**A**): CA conditioning arena, UA unpaired arena. Behavioral freezing response 24 h after (Post-US) conditioning session (Pre-US) with 1, 0.5, 0.25, and 0.15 mA footshock (**B**). Animals conditioned 10 days after 2 h of restraint stress did not show higher freezing levels comparing to unstressed animals regardless of the intensity of the US. Results are represented as mean ± SEM (*n* = 5 animals per group, 0.25 mA; *n* = 6 per group, 1 and 0.15 mA; *n* = 8 per group, 0.5 mA). Two-way ANOVA followed by Tukey’s multiple comparisons test. Significance differences between groups are indicated as *p < 0.05, **p < 0.01, ***p < 0.001, and ****p < 0.0001; n.s. nonsignificant.

The experimental design is illustrated in Fig. 1A. After the habituation period, half of the animals were randomly transferred to EE (where they remained for 14 consecutive days, pair-housed), and the other half remained pair-housed in standard cages until the end of the experiment. On the last EE day, half of both the EE and non-EE animals were restraint-stressed in the experimental room for 2 h, while the other half remained in their cages, undisturbed. Immediately after the stress session, the stressed and non-stressed animals (SC and EE) were transferred back to standard cages with the same previous cage mate. All groups remained on standard cages undisturbed, except for cage cleaning, for 10 consecutive days. After this interval, animals were assigned to the contextual fear conditioning paradigm, followed by retrieval tests or extinction sessions.

### Environmental enrichment

EE was conducted as described in ref. ^2^. The EE arena consisted of polypropylene cages (30 × 45 × 25 cm) in which we introduced several types of objects that were moved, replaced, and cleaned regularly (every 2 days), among them plastic balls and toys, wooden objects, metal and plastic ladders, hanging rope ladders, wooden boxes, PVC tunnels, and plastic and metal platforms of different sizes. The objects were introduced into the cage according to a previously established schedule at 3 to 4 at a time. Regular wood bedding covered the floor of all arenas.

### Acute restraint stress

The restrainer apparatus consisted of an opaque ventilated PVC cylinder (20 × 6 cm) with one end closed. The rats were transferred to the experimental room at 09:00 a.m. and restrained for a period of 2 h under constant monitoring.

### Contextual fear conditioning and extinction

Two different arenas were used to carry out the contextual fear conditioning and extinction: a conditioning chamber (conditioning arena, CA) and a neutral arena (unpaired arena, UA), both placed in the experimental room with the same temperature and luminosity. The CA (28 × 26 × 23 cm) consisted of opaque white walls, a transparent lid, and stainless-steel bars on the floor connected to an electric shock generator (Insight Equipamentos, Pesquisa e Ensino, Ribeirão Preto, SP, Brazil). The UA presented black opaque plastic walls on each side and floor, and a transparent lid (21 × 26 × 27.5 cm). On the training session (10 days after the acute restraint stress), each animal was individually placed into the CA and allowed to freely explore for 2 min before unconditioned stimulus (US) presentation (1 electrical footshock of 1, 0.5, or 0.3 mA, 1 s). The animals returned to their home cages 30 s after the end of the US. For the retrieval test, 24 h after training, the animals were individually placed back into the CA, where they remained for 5 min in the absence of the US. For the extinction paradigm, 24 h after the training session, the animals returned to the CA for a 10-min session with no US presentation. This procedure was repeated for four consecutive days in five trial sessions, with a 24-h intertrial interval. Twenty-four hours after the last extinction session, the animals were placed back to the CA for a 5-min-extinction test (Fig. 1A).

To attest the pairing US-context, part of the animals was placed in the UA 24 h after training and let to freely explore it for 10 min, followed by four more exposures with 24 h intervals. Twenty-four hours after the fifth trial, the animals returned to CA (paired) (Fig. 1A). This control was to verify whether changes in animal behavior during fear extinction were related to time-lapsing or the re-exposure to the CA in the absence of the US.

We videotaped the rats’ behavior using a digital video camera (Logitech C920, Lausanne, Switzerland) from the top of the chamber, and the freezing response, used to measure fear memory, was scored automatically on ANY-Maze software (4.99 m version, Stoelting, IL, USA). We considered freezing as complete immobility of the animal (including vibrissae and sniffing movements) except for respiration-related movements^14^. Freezing was analyzed in the training session (2 min before the US presentation; Pre-US) and the first and last 5 min of each trial, except for the trials that lasted 5 min, in which the freezing analysis was performed just in one block. After each trial, the chambers were cleaned with 5% alcohol to avoid olfactory cues. All behavioral tests were conducted between 09:00 a.m. and 12:00 p.m.

### Euthanasia and sample preparation

#### Decapitation and sample collection

The animals were deeply anesthetized with isoflurane (maximal exposure of 30 s) and decapitated with a guillotine (Insight Equipamentos, Pesquisa e Ensino, Ribeirão Preto, SP). The brain was rapidly removed from the skull in phosphate-saline buffer solution (137 mM NaCl, 2.68 mM KCl, 1.27 mM KH_2_PO_4_, 8.06 mM Na_2_HPO_4_). The HC, BLA, and FC (frontal cortex) were bilaterally dissected on ice, frozen in a conical tube (1.5 ml) on dry ice, and stored at −80 °C for later use. For BLA’s dissection, the brains were placed in a coronal matrix with 1 mm slice intervals (Zivic Laboratories Inc., PA, USA), and the dissection was performed under a stereomicroscope (Tecnival–Prolab, São Paulo, SP). The trunk blood was collected at the time of decapitation in a falcon tube (15 ml).

#### Protein extraction

HC, BLA, and FC tissues stored at −80 °C were thawed, mechanically homogenized with a conical plastic pestle (Thermo Fisher Scientific, MA, USA) in RIPA buffer (50 mM Tris-HCl, 1% NP-40, 0.25% Na-deoxycholate, 150 mM NaCl, 1 mM EDTA) supplemented with protease and phosphatase inhibitors (Halt Protease Inhibitor Cocktail, Thermo Fisher Scientific), and incubated for 10 min on ice. Then, the homogenates were centrifuged at 10,000 r.p.m. for 10 min at 4 °C. The supernatant containing the protein extract was collected in a tube and stored at −80 °C. Protein concentration was determined using the Bradford method (Bio-Rad Laboratories, Inc.).

### Western blot

The proteins were adjusted in the concentration of 2 μg/μl with Laemmli’s buffer (Bio-Rad Laboratories, Inc.; complemented with 5% mercaptoethanol) and boiled for 5 min at 95 °C. Electrophoresis was performed using the Mini-Protean® Tetra Cell apparatus (Bio-Rad Laboratories, Inc).

Protein samples (20 μg/lane) were size-separated in 10% SDS-PAGE gel (90 V) and then blotted onto Immobilon® PVDF membrane (EMD Millipore Corporation). The Ponceau method to immunoblot was used to ensure equal protein loading^15^. Blots were blocked with 5% bovine serum albumin (BSA) diluted in TBS-T buffer (50 mM Tris-HCl, 150 mM NaCl, 0.1% Tween 20, pH 7.5) for 1 h at room temperature, and subsequently incubated overnight at 4 °C with specific primary antibodies: polyclonal rabbit anti-GluA1 (AB1504, dilution 1:2000; EMD Millipore Corporation), polyclonal rabbit anti-Ser845phospho-GluA1 (AB5849, dilution 1:1000; EMD Millipore Corporation), polyclonal rabbit anti-GluN2B (#454582, dilution 1:1000; EMD Millipore Corporation), polyclonal rabbit anti-GluN2C (#454584, dilution 1:1000; EMD Millipore Corporation), monoclonal mouse anti-synaptophysin (AB8049, dilution of 1:20,000; Sigma-Aldrich Corporation), and monoclonal mouse anti-PSD-95 (AB99099, dilution of 1:20,000; Abcam). After incubation with the primary antibodies, the membrane was then probed with a secondary antibody conjugated to horseradish peroxidase (dilution of 1:2000; Kirkegaard & Perry Laboratories) for 2 h at room temperature and the signal was obtained by the Luminata Forte Western HRP substrate (Merck Millipore, Darmstadt, Germany) using the ChemiDoc system (Bio-Rad Laboratories). Several exposure times were performed to ensure the linearity of the band intensities. Each band’s relative density was normalized to the value of α-tubulin (sc-5286, dilution: 1:20,000; Santa Cruz Biotechnology).

### Statistical analysis

Statistical analysis was performed using two-way ANOVA, in which housing conditions (standard or EE) and stress (unexposed or exposed) were factors. Post hoc Tukey’s multiple comparison test examined differences between individual groups. Sample size and animal numbers were estimated based on previous studies and outliers were checked by the ROUT method. Data analysis and quantification were blind for all experiments. GraphPad Prism software 7.0 was used for the statistical analysis, and the level of statistical significance was set up at *p* < 0.05. Data are presented as mean ± SEM.

## Results

### Late effects of acute restraint stress on the acquisition of contextual fear memory

We first investigated whether a previous single exposition to stress influenced the acquisition of contextual fear conditioning. Animals were trained in the fear conditioning paradigm 10 days after been submitted to 2 h of acute restraint stress and were re-exposed to the same conditioning context 24 h later. Two-way ANOVA showed no main effect in the percentage of freezing to the stress factor in animals whose training was applied 1 mA of footshock [*F*(1, 30) = 3.845, *p* = 0.0592]. The increased freezing level observed in the animals during the retrieval test (Post-US) compared to the training session (Pre-US), confirmed by Tukey’s post hoc test (Fig. 1B), indicates that both stressed and non-stressed animals formed long-term memory [two-way ANOVA, *F*(1, 30) = 11.64, *p* < 0.0001; test session factor]. Assuming that the possibility of unconditioned stimulation (1 mA of footshock) was too aversive and generated a ceiling effect in the freezing response, which would have masked any differences between the control and stressed animals, we did three more training batteries using gradually lower footshock intensities. Two-way ANOVA showed a significant main effect of the test session (training × retrieval) [*F*(1, 34) = 153.54, *p* < 0.0001, for 0.5 mA; *F*(1, 20) = 37.08, *p* < 0.0001 for 0.25 mA; *F*(1, 30) = 36.97, *p* < 0.0001, for 0.15 mA]. As shown in Fig. 1B, Tukey’s multiple comparisons test confirmed that the animals trained with lower footshock intensities (0.5, 0.25, and 0.15 mA) were still able to form long-term memory, indicated by the significant increase of freezing during the retrieval test compared to the training session. Nonetheless, two-way ANOVA showed no main effect in the level of freezing to the stress factor [*F*(1, 34) = 0.2623, *p* = 0.6118, for 0.5 mA; *F*(1, 20) = 0.2983, *p* = 0.5910, for 0.25 mA; *F*(1, 30) = 1.767, *p* = 0.1937, for 0.15 mA].

### Previous EE prevents the late effect of acute restraint stress-induced fear extinction impairment

**Fig. 2:**
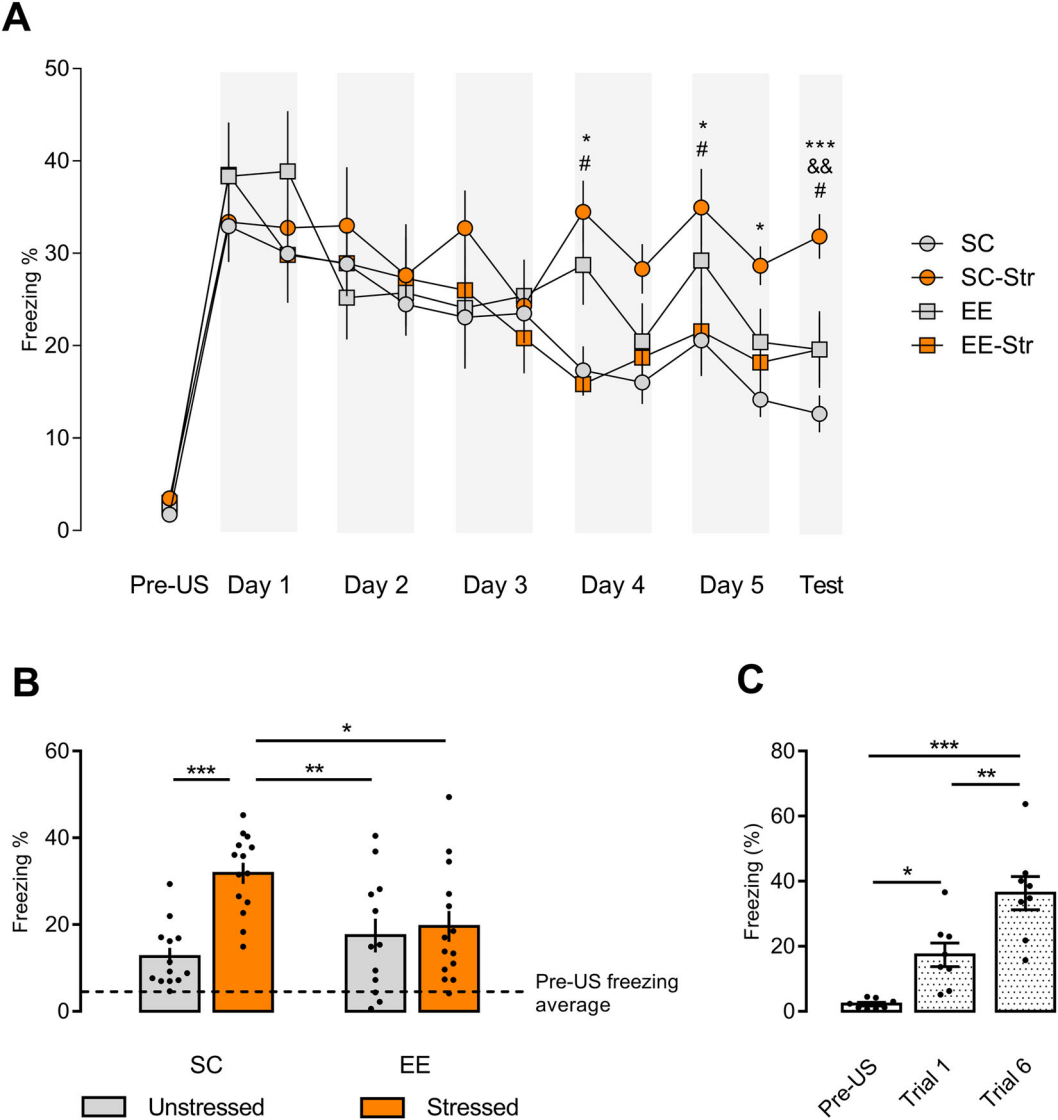
EE protects against acute restraint stress effect on late fear extinction impairment. Stressed SC-housed animals (SC-Str) showed a higher percentage of freezing comparing to SC-housed, unstressed (SC) and EE-housed, stressed (EE-Str) rats on the days 4, 5 (during the initial 5 min of test session), and 6, and higher than EE-housed, unstressed animals (EE) on day 6 post-acquisition training (**A**). The column graph depicts the higher behavioral freezing response of SC-Str animals comparing to SC and EE (EE and EE-Str) rats on the extinction test (**B**). Animals placed on the unpaired arena (UA) showed a higher percentage of freezing during trial 1 than during acquisition training (Pre-US), but lower than during trial 6, when animals were placed back on the paired arena (**C**). Results are presented as mean ± SEM (*n* = 8–14 animals per group in **A**; *n* = 14 per group in **B**; *n* = 8 per group in **C**). Two-way ANOVA (**A**, **B**) and one-way ANOVA (**C**) followed by Tukey’s multiple comparisons test. Significance differences between groups are indicated as *, **, and *** in relation to SC (**A**, **B**) or in relation to Pre-US (**C**) (*p* < 0.05, *p* < 0.01, and *p* < 0.001, respectively); # in relation to EE-Str (**A**, *p* < 0.05); && in relation to EE (**A**, *p* < 0.01).

Once there were no effects of stress on the fear acquisition, regardless of the US intensity, we next decided to use an intermediate footshock intensity (0.5 mA) to investigate whether a previous stress event could influence, 10 days after, the extinction of the acquired aversive memory. As presented in Fig. 2, acute restraint stress, given 10 days before the fear conditioning training, impaired the contextual fear extinction in standard cage-housed animals (SC-Str) since their percentage of freezing on day 1 (24 h after the training session) was not different from the other consecutive trials [one-way ANOVA, *F*(5, 46) = 0.1033, *p* = 0.9910]. Nevertheless, non-stressed animals housed in standard cages (SC) decreased freezing response after successive re-exposure to the paired context [one-way ANOVA, *F*(5, 53) = 5.158, *p* = 0.0006; confirmed by Tukey’s multiple comparisons test] (Fig. 2A). Considering the percentage of freezing in the extinction test (sixth day), two-way ANOVA revealed significant main effects of stress exposure [*F*(1, 49) = 12.23, *p* = 0.010] and stress-housing conditions interaction [*F*(1, 49) = 7.866, *p* = 0.0072], with no effect of housing [*F*(1, 49) = 1.468, *p* = 0.2315] (Fig. 2A, B). Tukey’s post hoc test confirmed that the freezing response was significantly higher in the SC-Str animals than that showed by SC animals (Fig. 2A, B). Additionally, Tukey’s post hoc test confirmed a protective role of EE on the stress-induced fear extinction impairment. EE, both in stressed (EE-Str) and non-stressed (EE) animals, did not change the percentage of freezing when compared to SC animals but was significantly lower than in SC-Str ones (Fig. 2A, B). This result suggests that previously EE housing prevents stress-induced impairment in contextual fear extinction but not increases the extinction process itself, confirming EE’s protective role on the stress-induced fear extinction impairment.

The animals submitted to the UA in trial 1 showed an increase in freezing response compared to the training period (Pre-US), but they showed a statistically significant increase in the freezing response when submitted to the paired arena in trial 6 [one-way ANOVA, *F*(2, 21) = 22.15, *p* < 0.0001; confirmed by Tukey’s multiple comparisons test] (Fig. 2C). These data indicate that the reduction in freezing response is not related to the time lapse between trial 1 and 6 but, instead, to the successive animal re-exposure to the paired arena.

### EE prevented the acute stress increase in pGluA1/GluA1 expression in the HC, but not in the BLA and FC, in animals that underwent fear conditioning/extinction paradigm

**Fig. 3:**
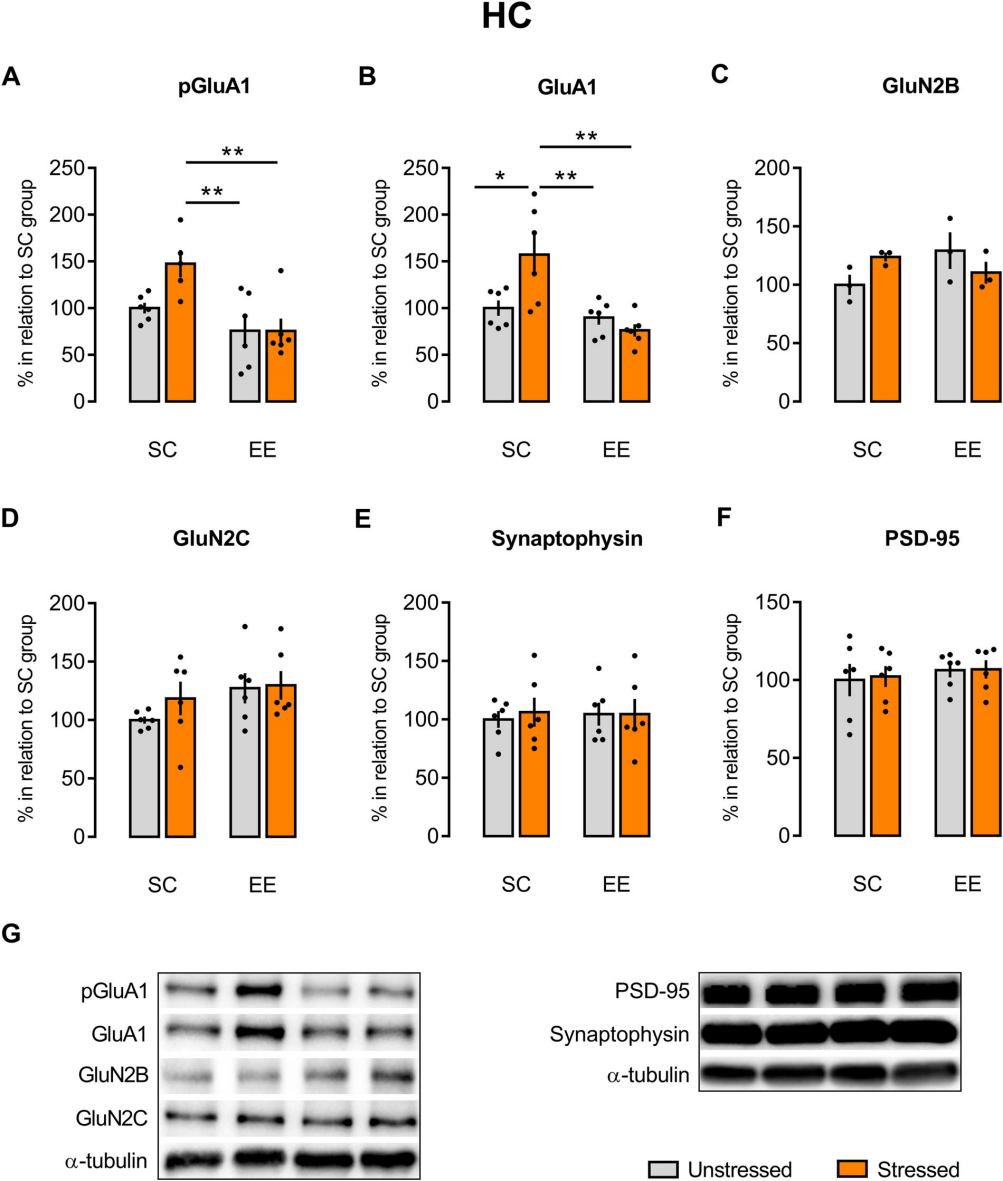
EE prevents the stress-induced increase in pGluA1/GluA1 expression in the HC. Stressed SC-housed animals showed higher expression of pGluA1 (**A**) and GluA1 (**B**) in the HC comparing to SC-housed, unstressed (only in **B**), EE-housed, stressed and unstressed animals. EE-housed animals (both stressed and unstressed) presented the same expression levels of pGluA1 (**A**) and GluA1 (**B**) than SC-housed, unstressed animals. There were no differences in the GluN2B (**C**) and GluN2C (**D**) expression in both SC- and EE-housed animals (stressed or unstressed). There were no differences in the synaptophysin (**E**) and PSD-95 (**F**) expression in both SC- and EE-housed animals, regardless of the presence of stress. Representative autoradiography of western blot (**G**). Results are represented as mean ± SEM (*n* = 5–6 animals per group in **A**; *n* = 6 for each group in **B**, **D**–**F**; *n* = 3 for each group in **C**). Two-way ANOVA followed by Tukey’s multiple comparisons test. Significance differences between groups are indicated as *p < 0.05 and **p < 0.01.

**Fig. 4:**
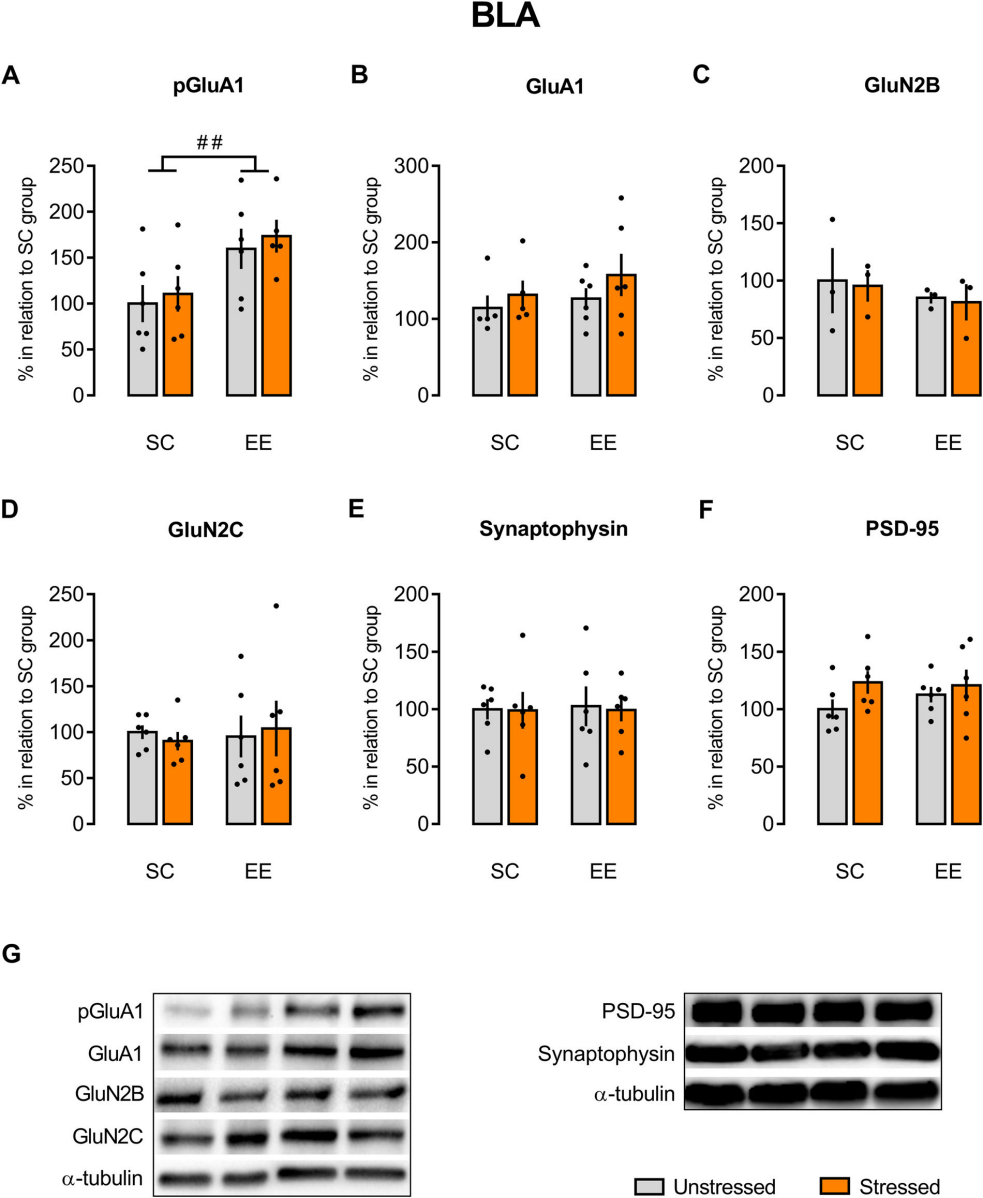
There is no stress effect on the GluA1 phosphorylation in the BLA, but there is an EE effect. There was an environmental effect on the pGluA1 (**A**) expression in the BLA, but there were no effects of either environment or stress on the expression of GluA1(**B**), GluN2B (**C**), GluN2C (**D**), synaptophysin (**E**), and PSD-95 (**F**) in the same brain area. Representative autoradiography of western blot (**G**). Results are represented as mean ± SEM (*n* = 5–6 animals per group in **A** and **B**; *n* = 6 for each group in **D**–**F**; *n* = 3 for each group in **C**). Two-way ANOVA followed by Tukey’s multiple comparisons test (no differences were detected between the experimental groups). ANOVA significant main effect is indicated as ^##^*p* < 0.01.

**Fig. 5:**
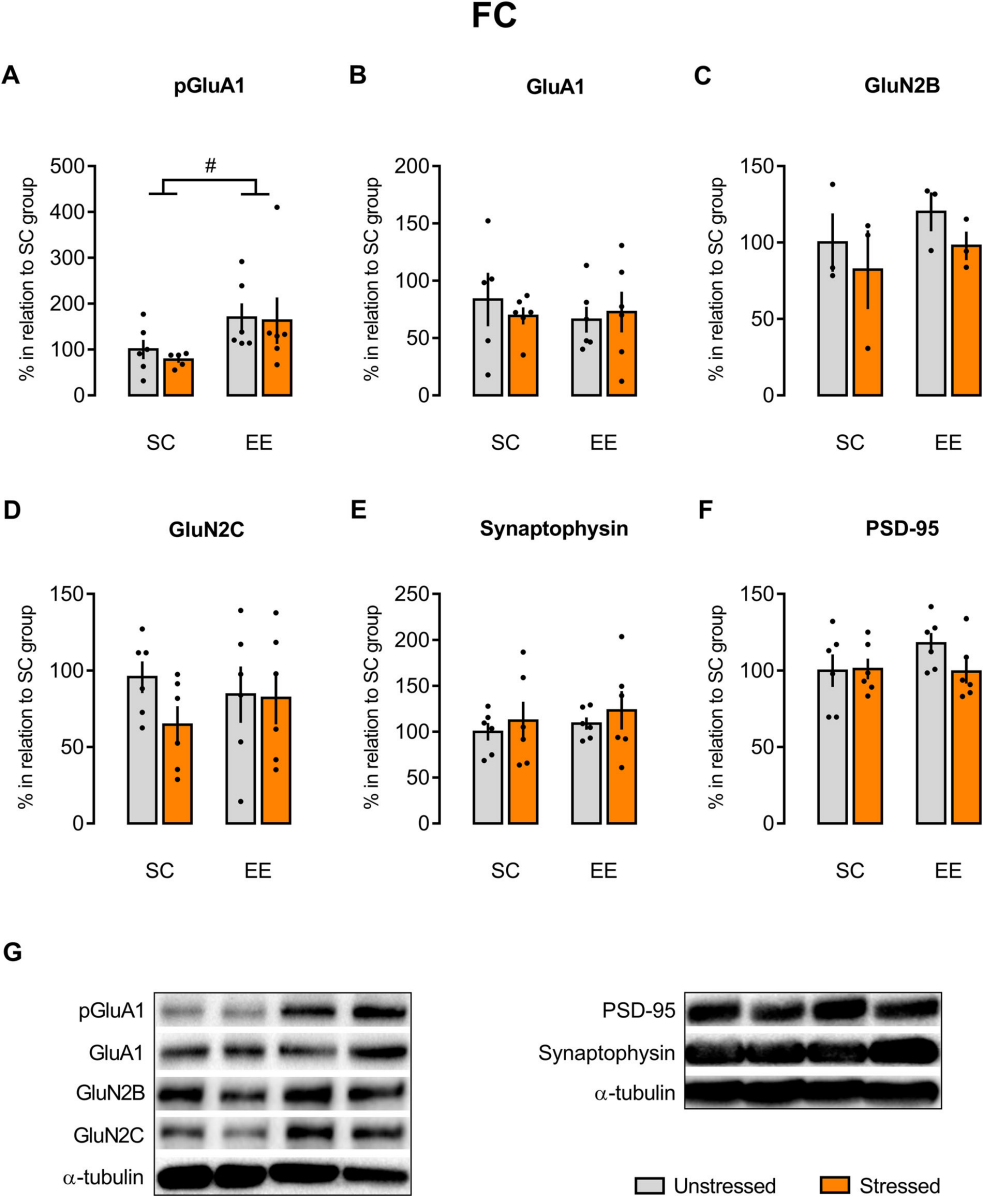
There is no stress effect on the GluA1 phosphorylation in the FC, but there is an EE effect. There was an environmental effect on the pGluA1 (**A**) expression in the FC, but there were no effects of either environment or stress on the expression of GluA1 (**B**), GluN2B (**C**), GluN2C (**D**), synaptophysin (**E**), and PSD-95 (**F**) in the same brain area. Representative autoradiography of western blot (**G**). Results are represented as mean ± SEM (*n* = 5–6 animals per group in **A** and **B**; *n* = 6 for each group in **D**–**F**; *n* = 3 for each group in **C**). Two-way ANOVA followed by Tukey’s multiple comparisons test (no differences were detected between the experimental groups). ANOVA significant main effect is indicated as ^#^*p* < 0.05.

Knowing the role of AMPA and NMDA receptors in the neuronal plasticity, we next checked the state of phosphorylation and expression of different AMPA and NMDA receptors subunits in the HC (Figs. 3 and S1), BLA (Figs. 4 and S2), and FC (Figs. 5 and S3) of the animals that underwent the fear extinction paradigm. Two-way ANOVA revealed main effects of housing [*F*(1, 20) = 13.13, *p* = 0.0017] and housing-stress interaction [*F*(1, 20) = 7.96, *p* = 0.0105] in the expression of AMPA-GluA1 and a main effect of housing [*F*(1, 19) = 13.68, *p* = 0.0015] in the expression of AMPA-Ser845phospho-GluA1 in the HC. As we can see in Fig. 3, Tukey’ multiple comparisons test confirmed that acute restraint stress promoted a late effect of Ser845phospho-GluA1 (Fig. 3A) and GluA1 (Fig. 3B) expression in the HC of SC-housed animals (SC-Str) comparing to EE (stressed or unstressed animals) and to SC, unstressed rats (only for GluA1). Moreover, there were no effects of stress, EE housing exposition, and stress–housing interaction in the HC NMDA-GluN2B [*F*(1, 8) = 0.05826, *p* = 0.8153; *F*(1, 8) = 0.591, *p* = 0.4641; *F*(1, 8) = 4.319, *p* = 0.0713, respectively] and NMDA-GluN2C [*F*(1, 20) = 0.821, *p* = 0.3757; *F*(1, 20) = 2.788, *p* = 0.1106; *F*(1, 20) = 0.4868, *p* = 0.4934, respectively] expression (Fig. 3C, D). Two-way ANOVA also showed no effects in the HC expression of the presynaptic marker synaptophysin [*F*(1, 20) = 0.01863, *p* = 0.8928 for housing exposition; *F*(1, 20) = 0.08358, *p* = 0.7755 for stress; *F*(1, 20) = 0.08162, *p* = 0.7781 for housing-stress interaction; Fig. 3E] and the pos-synaptic marker PSD-95 [*F*(1, 20) = 0.5787, *p* = 0.4557 for housing exposition; *F*(1, 20) = 0.03878, *p* = 0.8459 for stress; *F*(1, 20) = 0.01577, *p* = 0.9013 for housing-stress interaction; Fig. 3F].

Regarding the BLA, two-way ANOVA showed a main effect of housing [*F*(1, 19) = 9.194, *p* = 0.0069], not confirmed by the Tukey’s post hoc test, and no main effects of stress [*F*(1, 19) = 0.3654, *p* = 0.5527] and housing–stress interaction [*F*(1, 19) = 0.006247, *p* = 0.9378] in the expression of Ser845phospho-GluA1 (Fig. 4A). With respect to GluA1 expression (Fig. 4B), two-way ANOVA showed no main effects of housing [*F*(1, 18) = 0.8772, *p* = 0.3614], stress [*F*(1, 18) = 1.393, *p* = 0.2533], and housing–stress interaction [*F*(1, 18) = 0.1105, *p* = 0.7434]. As seen in the HC, two-way ANOVA revealed no main effects of stress, housing exposition, and stress–housing interaction in the BLA expression of NMDA-GluN2B [*F*(1, 8) = 0.05697, *p* = 0.8173; *F*(1, 8) = 0.6725, *p* = 0.4359; *F*(1, 8) = 0.00034, *p* = 0.9857, respectively; Fig. 4C], NMDA-GluN2C [*F*(1, 20) = 0.00047, *p* = 0.9828; *F*(1, 20) = 0.05219, *p* = 0.8216; *F*(1, 20) = 0.2146, *p* = 0.6482, respectively; Fig. 4D], synaptophysin [*F*(1, 20) = 0.02333, *p* = 0.8801; *F*(1, 20) = 0.01242, *p* = 0.9124; *F*(1, 20) = 0.006967, *p* = 0.9343, respectively; Fig. 4E], and PSD-95 [*F*(1, 20) = 2.462, *p* = 0.1323; *F*(1, 20) = 0.2532, *p* = 0.6203; *F*(1, 20) = 0.5743, *p* = 0.4574, respectively; Fig. 4F].

With respect to the FC, we found quite similar results to those seen in the BLA. Two-way ANOVA showed a main effect of housing [*F*(1, 19) = 5.371, *p* = 0.0318], not confirmed by the Tukey’s post hoc test, and no main effects of stress [*F*(1, 19) = 0.1816, *p* = 0.6748] and housing–stress interaction [*F*(1, 19) = 0.0536, *p* = 0,8194] in the expression of Ser845phospho-GluA1 (Fig. 5A). Two-way ANOVA showed no main effects of housing [*F*(1, 19) = 0.2147, *p* = 0.6484], stress [*F*(1, 19) = 0.05807, *p* = 0.8122], and housing–stress interaction [*F*(1, 19) = 0.4589, *p* = 0.5063] in the GluA1 expression (Fig. 5B). Correspondingly, two-way ANOVA revealed no main effects of stress, housing exposition, and stress–housing interaction in the FC expression of NMDA-GluN2B [*F*(1, 8) = 1.254, *p* = 0.2953; *F*(1, 8) = 1.001, *p* = 0.3463; *F*(1, 8) = 0.01582, *p* = 0.9030, respectively; Fig. 5C], NMDA-GluN2C [*F*(1, 20) = 1.25, *p* = 0.2768; *F*(1, 20) = 0.04247, *p* = 0.8388; *F*(1, 20) = 0.9448, *p* = 0.3427, respectively; Fig. 5D], synaptophysin [*F*(1, 20) = 0.7091, *p* = 0.4097; *F*(1, 20) = 0.4034, *p* = 0.5325; *F*(1, 20) = 0.005736, *p* = 0.9404, respectively; Fig. 5E], and PSD-95 [*F*(1, 20) = 1.125, *p* = 0.3014; *F*(1, 20) = 0.9725, *p* = 0.3358; *F*(1, 20) = 1.431, *p* = 0.2456, respectively; Fig. 5F].

## Discussion

Ample evidence reveals a positive correlation between the manifestation of anxiety-like behavior and the extinction impairment of tone and contextual fear conditioning in rodents^16–18^. Indeed, patients with trauma-related disorders, such as PTSD, exhibit aversive memory extinction deficit, thought to be the genesis of maintenance of anxiety-related symptoms^19–22^. Correspondently, extinction learning is widely used as a model of cognitive-behavioral therapy^23^. The long-lasting anxiety-like behavior promoted by 2 h of restraint stress in rats is well-known^1,2,24^, but for the first time, the present findings show that the same stress also impaired fear extinction with no effects on memory acquisition. Even more impressive, the EE, in its turn, can prevent not only the late onset of anxiety, as we presented in our previous work^2^, but here we showed that it also prevented the deleterious effect of stress on the fear extinction process.

The effects of stress on memory acquisition and extinction are debatable. Some reports show that an acute footshock stress protocol (15 electrical shocks delivered over a 90-min interval) increased the conditioned fear memory acquisition and impaired the extinction to tone and context in animals trained 1 week after stress^25^. Others showed that the single prolonged stress paradigm (2 h restraint, 20 min of forced swimming, and deep sedation by ether inhalation) impaired the extinction of the tone and contextual conditioned fear memory in rats trained 1 week after stress^26–28^, with no effects on memory acquisition^26,28^. Our results, therefore, reinforce the findings on the late effect of acute stress on the extinction of the contextual fear memory with no effects on memory acquisition, even though the stress we used was less intense and the interval between stress and conditioning was longer.

Extinction of conditioned fear is considered a new learning event that inhibits the fear response to the conditioned stimulus^6,7^. The synapse strengthening during LTP induction is critically dependent on the trafficking of AMPA receptors to the synaptic membrane and the phosphorylation of the Ser831 and/or Ser845 residues of the GluA1 receptor subunit promotes the receptor cell-surface insertion^29–32^. AMPA receptors in adult rat neurons are predominantly combinations of GluA1, GluA2, and GluA3 subunits^33,34^. The long-tailed GluA1-containing AMPA receptors are rapidly mobilized from the endoplasmic reticulum to the synaptic membrane and are later replaced by the short-tailed GluA2/GluA3 subunits^29,35^.

Consolidation, retrieval, and extinction of conditioned fear are crucially dependent on contextual information encoded through hippocampal circuitry and an increase in the hippocampal GluA1 expression during consolidation and retrieval of fear memory is observed^31,36^. Another study showed that the fear extinction impairment promoted by the ethanol consumption was accompanied by the increase in the phosphorylation of GluA1 in the same brain structure^11^. Our data indicate not only a correlation between the increase in the levels of expression and phosphorylation of GluA1 into the HC and the acute stress-induced late fear extinction impairment (with no alterations in the expression of GluN2B and GluN2C NMDA subunit receptors) but also the preventive effect of EE on the behavioral outcome of stress and its capacity to damp the stress-induced molecular alterations in this AMPA receptor subunit.

BLA is also an essential structure for fear memory acquisition and extinction, phenomena that are dependent on glutamate-mediated synaptic plasticity^8,37,38^. Fear memory acquisition demands an increase of GluA1-containing AMPA receptors insertion in the synaptic membrane, whereas the extinction of the memory is characterized by the reversion of this process, attributed to the internalization of those receptors^8,32,39^. Accordingly, before extinction sessions, AMPA antagonism in the BLA does not alter the efficiency of the contextual fear memory extinction^40^.

The dense projections from the medial prefrontal cortex (mPFC) to the BLA, resulting in inhibitory control over amygdalar throughput, are considered critical for fear extinction^41^. Changes in synaptic plasticity and LTP in this brain region, therefore, influence the extinction process. Administration of the AMPA receptor agonist PEPA in the mPFC facilitates the extinction of contextual fear memory and promotes an increase in AMPA receptors in synaptic terminals^42^. Although our data are related to the FC, i.e., it is not restricted to mPFC but includes it; they corroborate these findings. Acute stress, which caused a late fear extinction deficit, did not promote changes in the GluA1 phosphorylation state in the FC, while EE, which exerted an extinction-facilitating effect, promoted an increase in GluA1 phosphorylation at the Ser845 residue.

Synaptophysin and PSD-95, markers of synaptic density, are present in excitatory synapses and changes in their expression indicate a synaptic remodeling process^43^. The basal expression of PSD-95 is a requisite for the appropriate synaptic plasticity mediated by AMPA receptors. It has been shown that phosphorylated GluA1-containing AMPA receptors require direct interaction with PSD-95 for trafficking and insertion in the synaptic membrane^29,44^. Nonetheless, our results showed that the increased phosphorylation of GluA1-AMPA receptor is not always accompanied by an increase in the expression of PSD-95, or synaptophysin, suggesting that under appropriate baseline expression, the plasticity of glutamatergic synapses can occur without detectable changes in these proteins. Noteworthy, while studies using chronic immobilization stress in mice have revealed a negative modulatory effect on the expression of synaptophysin in the amygdala and HC^45,46^, long-term EE protocols (at least 4 weeks of duration), on the other hand, are related to increased synaptophysin and PSD-95 in HC and FC^47–49^. Considering our findings, both stress and EE’s temporal component is noticeable on the modulatory effect on these synaptic markers’ expression.

Our results indicate that the increased phosphorylation at the Ser845 residue of the GluA1-AMPA receptor in the HC, suggestive of increased trafficking of this receptor to the synaptic membrane, is associated with the late impairment of fear memory extinction triggered by acute stress. In contrast, the increase in the phosphorylation of Ser845-GluA1-AMPA receptors in the BLA and FC are related to the EE-induced protective effect on fear extinction in the face of a stressor stimulus. Interestingly, some authors suggest that the global blockade of AMPA internalization (keeping the receptor in the synaptic membrane) in the brain during the memory extinction disrupts the recall of extinction memory, as opposed to the same blockage during the fear conditioning and memory recall not affecting the expression of fear^50^.

In summary, although some studies using different types of stress have already shown the persistent effects of stress directly modulating the extinction of fear memory, the present study was the first to show that EE before the stress event can prevent the emergence of stress-related extinction impairment, pointing out a possible molecular mechanism through which the EE is protective. Altogether, these findings further contribute to understanding how stress-associated behavioral disorders are established, suggesting a target that can be modulated by a non-pharmacological approach.

## Supplementary information

Suplementary Figure S1.

Suplementary Figure S2.

Suplementary Figure S3.
