# Supplementary figures and images for: Environmental enrichment prevents the late effect of acute stress-induced fear extinction deficit: the role of hippocampal AMPA-GluA1 phosphorylation
Source: Transl Psychiatry. 2021 Jan 5;11:18. doi: 10.1038/s41398-020-01140-6 (PMC7791025; doi:10.1038/s41398-020-01140-6)

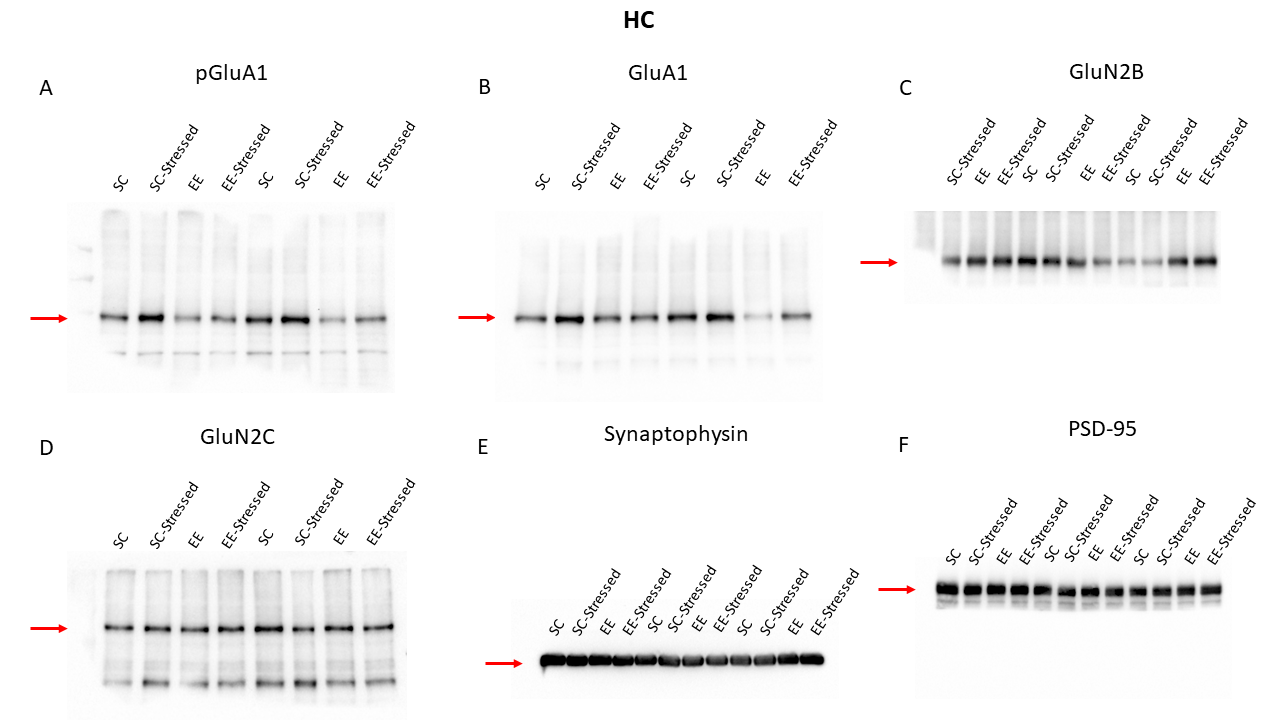

Supplement: Supplementary file 1 — Suplementary Figure S1. [file 41398_2020_1140_MOESM1_ESM.tif]

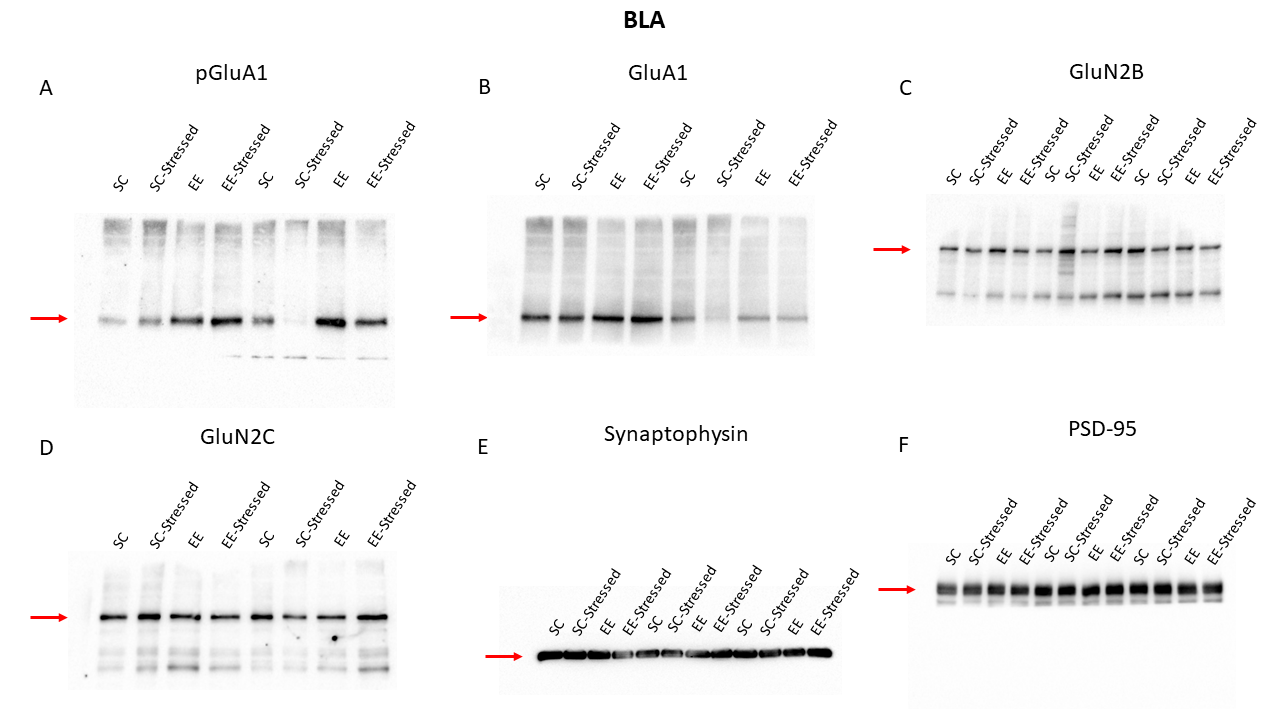

Supplement: Supplementary file 2 — Suplementary Figure S2. [file 41398_2020_1140_MOESM2_ESM.tif]

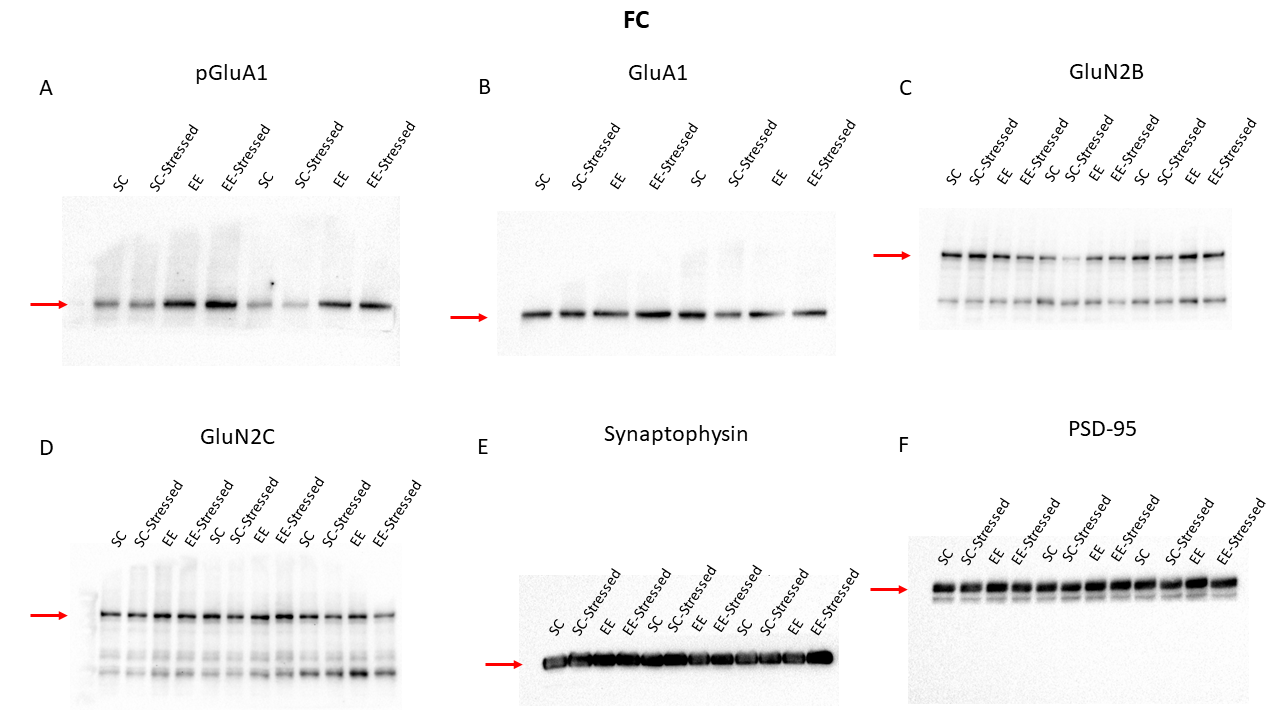

Supplement: Supplementary file 3 — Suplementary Figure S3. [file 41398_2020_1140_MOESM3_ESM.tif]
